# Optimisation, harmonisation and standardisation of the direct mycobacterial growth inhibition assay using cryopreserved human peripheral blood mononuclear cells

**DOI:** 10.1016/j.jim.2019.01.006

**Published:** 2019-06

**Authors:** Rachel Tanner, Steven G. Smith, Krista E. van Meijgaarden, Federico Giannoni, Morven Wilkie, Lucia Gabriele, Carla Palma, Hazel M. Dockrell, Tom H.M. Ottenhoff, Helen McShane

**Affiliations:** a The Jenner Institute, University of Oxford, UK; b Department of Immunology and Infection, London School of Hygiene and Tropical Medicine, UK; c Department of Infectious Diseases, Leiden University Medical Centre, Leiden, The Netherlands; d Department of Infectious Diseases, Istituto Superiore di Sanità, Italy; e Department of Oncology and Molecular Medicine, Istituto Superiore di Sanità, Italy

**Keywords:** Tuberculosis, Vaccine, Mycobacterial growth inhibition assay, MGIA, Harmonisation

## Abstract

A major challenge to tuberculosis (TB) vaccine development is the lack of a validated immune correlate of protection. Mycobacterial growth inhibition assays (MGIAs) represent an unbiased measure of the ability to control mycobacterial growth *in vitro.* A successful MGIA could be applied to preclinical and clinical post-vaccination samples to aid in the selection of novel vaccine candidates at an early stage and provide a relevant measure of immunogenicity and protection. However, assay harmonisation is critical to ensure that comparable information can be extracted from different vaccine studies. As part of the FP7 European Research Infrastructures for Poverty Related Diseases (EURIPRED) consortium, we aimed to optimise the direct MGIA, assess repeatability and reproducibility, and harmonise the assay across different laboratories. We observed an improvement in repeatability with increased cell number and increased mycobacterial input. Furthermore, we determined that co-culturing in static 48-well plates compared with rotating 2 ml tubes resulted in a 23% increase in cell viability and a 500-fold increase in interferon-gamma (IFN-γ) production on average, as well as improved reproducibility between replicates, assay runs and sites. Applying the optimised conditions, we report repeatability to be <5% coefficient of variation (CV), intermediate precision to be <20% CV, and inter-site reproducibility to be <30% CV; levels within acceptable limits for a functional cell-based assay. Using relevant clinical samples, we demonstrated comparable results across two shared sample sets at three sites. Based on these findings, we have established a standardised operating procedure (SOP) for the use of the direct PBMC MGIA in TB vaccine development.

## Highlights

• Optimised assay conditions for direct PBMC MGIA determined.
• Standardised operating procedure for use in TB vaccine studies established.
• Consistency across three sites using clinically-relevant samples demonstrated.

## 1 Introduction

Tuberculosis (TB) is a major global health problem, with 10 million new cases and 1.6 million deaths in 2017 (WHO, 2018). BCG, the only currently available TB vaccine, has poor efficacy against adult pulmonary disease in the tropics, where TB incidence is greatest (Colditz et al., 1994). Given the high rates of infection, insufficiency of BCG and the rising threat from drug-resistant TB (WHO, 2018), an effective vaccine is urgently required. However, vaccine development is hampered by the lack of a validated immune correlate of protection from TB (Ottenhoff et al., 2012). Such a correlate or biomarker would aid in the selection of novel vaccine candidates at an early stage and provide a relevant measure of immunogenicity in phase I trials. In the absence of such a biomarker, the protective potential of a vaccine can only be determined by comparing it to BCG in costly large-scale, long-term phase III field efficacy trials (Ottenhoff et al., 2012).

Mycobacterial growth inhibition assays (MGIAs) represent an alternative to single biomarkers based on predefined individual immune parameters; instead offering an unbiased measure of the functional ability of post-vaccination whole blood or cell samples to control mycobacterial growth *in vitro* (Tanner et al., 2016)*.* A successful MGIA ie. one that is validated against clinical outcomes such as protection against natural or experimental mycobacterial infection or disease, would greatly facilitate TB vaccine development, decreasing the time and resources required for preclinical evaluation. It may also aid in the selection of candidates going forward to preclinical virulent *M.tb* challenge experiments, in line with the principles of the 3Rs (Replacing, Reducing and Refining the use of animals in scientific research) (Tanner and McShane, 2016). Once biologically validated in preclinical models, bridging to the use of human samples would allow evaluation of protective potential in the target species. Furthermore, MGIAs offer a tractable model for the investigation of immune mechanisms underlying mycobacterial control (de Vallière et al., 2005; Smith et al., 2016; Chen et al., 2016; Tanner et al., 2016, 2017; Joosten et al., 2018).

Several MGIAs have been reported in the literature (Worku and Hoft, 2000; Cheon et al., 2002; Kampmann et al., 2004; Parra et al., 2009; Fletcher et al., 2013; Tanner et al., 2016; Joosten et al., 2018) and optimisation and harmonisation efforts have been made, including the formation of working groups and publication of protocols (Zelmer et al., 2015; Brennan et al., 2017; Kaufmann et al., 2017). However, such assays are technically demanding and to date none has been systematically assessed for reproducibility or standardised and validated across laboratories for use in TB vaccine development. Assay harmonisation is critical to ensure that comparable information can be extracted from ongoing and future preclinical and clinical vaccine trials conducted across different organisations and settings, and with different vaccine candidates. Tuomela et al. (2005) describe precision as a fundamental assay validation parameter, encompassing three levels: repeatability, intermediate precision and reproducibility (Tuomela et al., 2005). Repeatability (intra-assay precision) measures the variation between multiple determinations of a single sample in a single test run, while intermediate (inter-assay) precision considers the variation between multiple measures of a single sample analysed in different assay runs. Reproducibility is concerned with the variation between multiple determinations of a single sample analysed at different laboratories or sites (Tuomela et al., 2005).

European Research Infrastructures for Poverty Related Diseases (EURIPRED) was a European Commission-funded Framework Program 7 consortium project that ran from 2013 to 2017. The overall goal was to coordinate and integrate international resources into a single specialised infrastructure to support European TB, HIV, Malaria and Hepatitis B/C studies from early drug, vaccine and microbicide discovery to clinical trials. EURIPRED was comprised of several work packages including resource management and quality assurance, sample selection and characterisation of biological materials, development and evaluation of reference reagents, and dissemination and training. Work package 5, standardisation and harmonisation of assays, aimed to select key immunological assays currently used in vaccine development, conduct harmonisation experiments and define standardised SOPs for dissemination.

We focussed on the optimisation and harmonisation of the direct MGIA, based on the methods of Wallis et al. (2001) (Wallis et al., 2001) and recently adapted for use in TB vaccine studies (Fletcher et al., 2013; Brennan et al., 2017). This MGIA was selected due to its relative simplicity, the use of standardised reagents and equipment, and advantages of the BACTEC MGIT quantification system over traditional colony counting (Brennan et al., 2017). Peripheral blood mononuclear cells (PBMC) were chosen as the use of cryopreserved cells aids in transferability to different trial sites, eliminating the need to evaluate samples in real time using fresh material and enabling direct comparison of pre- and post-vaccination samples in a single experiment. Optimisation of various assay parameters was conducted, including multiplicity of infection (MOI), lysis reagents and co-culture methodology. Outcomes for shared sample sets were compared across three different laboratories and repeatability, intermediate precision and reproducibility were assessed under different assay conditions. Based on these findings, a standardised SOP was defined and applied to shared sets of clinically relevant samples.

## 2 Methods

### 2.1 Human samples

Samples used in the optimisation experiments were obtained from local blood transfusion services as buffy coats. PBMC were processed and cryopreserved at one site and vials distributed to the other three laboratories.

The phase of the project in which the assay was applied to clinically relevant samples used either PBMC from healthy Dutch donors enrolled in a BCG vaccination study that was approved with protocol P12.087 (Boer et al., 2015), or PBMC from healthy UK adults aged 18–55 from a BCG vaccination study that was approved by the Oxfordshire Research Ethics Committee (C00.219) (McShane et al., 2004). Samples were also obtained from healthy adult volunteers at the London School of Hygiene and Tropical Medicine who were either BCG naïve or historically vaccinated, and who gave informed, written consent. The Ethics Committee of the London School of Hygiene and Tropical Medicine (ref. 5520) gave approval for the use of these samples. Samples for the final harmonisation experiment were taken from a study of BCG vaccination in healthy UK adults (NCT02380508) conducted at the Jenner Institute, University of Oxford, which was approved by NRES Committee South Central - Oxford B (REC ref. 15/SC/0022). Volunteers were aged between 18 and 45 years with no evidence of latent *M.tb* infection. Individuals received a single intradermal dose of 2-8 × 10^5^ CFU of BCG SSI and bloods were taken pre-vaccination at day 0, and days 2, 4, 7, 10, 14, 21, 28 and 84 post-vaccination.

All methods used in the clinical trials described were carried out in accordance with the ethical principles set forth in the Declaration of Helsinki as agreed by the World Medical Association General Assembly (Washington 2002), ICH Good Clinical Practice (GCP) and local regulatory requirements. All volunteers gave informed consent after the nature and possible consequences of the studies had been fully explained.

### 2.2 Sample processing

PBMC were isolated from buffy coat or heparinised peripheral blood samples using density centrifugation. Cells were counted, resuspended in foetal bovine serum (FBS) and incubated at 4 °C for 30 min. An equal volume of FBS containing 20% dimethylsufoxide was then added and cells aliquoted at a concentration of 5–10 × 10^6^ cells per cryovial, frozen overnight at −80 °C and transferred to liquid nitrogen storage the following day. Cryopreserved PBMC were thawed in a water bath at 37 °C until a small amount of frozen material remained. Samples were gradually added to 10 ml RPMI (containing 10% FBS and 2 mM l-glutamine) using a Pasteur pipette. The cryovial was rinsed using 1 ml of fresh medium and added to the corresponding tube, which was then centrifuged at 1500 rpm for 5 min. Supernatants were poured off and cells resuspended at an approximate concentration of 2–3 × 10^6^ cells per ml of RPMI (containing 10% foetal calf serum and 2 mM l-glutamine), and 2 μl/ml of 25 U benzonase (final concentration 50 U/ml) added to each tube. Cells were rested at 37 °C for 2 h with 5% CO_2_ before counting.

### 2.3 Direct PBMC MGIA

#### 2.3.1 Mycobacterial stock preparation

In-house BCG Pasteur stocks were grown in Middlebrook 7H9 medium with 10% OADC to mid-log phase then divided into 1 ml aliquots and stored at −80 °C until required. Aliquots were thawed at room temperature immediately prior to inoculation and diluted to the correct concentration (to give a final assay concentration of 500 CFU per culture) in RPMI (containing 2 mM l-glutamine and 25 mM HEPES). For harmonisation experiments, frozen aliquots of a single batch of BCG Pasteur stock (provided by Aeras, Rockville, USA) grown in Middlebrook 7H9 medium with 10% OADC and 0.05% tyloxapol were circulated and serially diluted prior to inoculation in a standardised way between sites.

#### 2.3.2 In-tube protocol

The in-tube direct PBMC MGIA was performed as previously described (Fletcher et al., 2013). Briefly, 2 ml screw-cap tubes containing 1 × 10^6^ PBMC and ~500 CFU BCG Pasteur (unless otherwise specified) in a total volume of 600 μl RPMI (containing 10% pooled human serum, 2 mM l-glutamine and 25 mM HEPES) were incubated on a 360° rotator (VWR International) at 37 °C for 96 h. Where specified, cells were lysed at the end of the culture period by centrifuging tubes at 12000 rpm for 10 min, removing supernatant and adding 500 μl sterile water (or other lysis agent as specified) to the pellet before pulse vortexing and transferring to BACTEC MGIT tubes supplemented with PANTA antibiotics (polymyxin B, amphotericin B, nalidixic acid, trimethoprim and azlocillin) and OADC enrichment broth (Becton Dickinson, UK). For experiments where cells were not lysed, cultures at the end of the 96 h period were transferred directly to supplemented BACTEC MGIT tubes.

#### 2.3.3 48-well plate protocol

48 well plates containing 3 × 10^6^ PBMC and ~500 CFU BCG Pasteur in a total volume of 600 μl RPMI (containing 10% pooled human serum or autologous serum where available and specified, 2 mM l-glutamine and 25 mM HEPES) per well were incubated at 37 °C for 96 h. At the end of the culture period, cultures were added to 2 ml screw-cap tubes and centrifuged at 12000 rpm for 10 min. During this time, 500 μl sterile water was added to each well to lyse adherent monocytes. Supernatants were removed from the 2 ml screw-cap tubes, and water from the corresponding well added to the pellet. Tubes were pulse vortexed and the lysate transferred to BACTEC MGIT tubes supplemented with PANTA antibiotics and OADC enrichment broth (Becton Dickinson, UK).

#### 2.3.4 Mycobacterial quantification

At the end of both protocols, tubes were placed on the BACTEC 960 machine (Becton Dickinson, UK) and incubated at 37 °C until the detection of positivity by fluorescence. On day 0, duplicate direct-to-MGIT viability control tubes were set up by inoculating supplemented BACTEC MGIT tubes with the same volume of mycobacteria as the samples. The time to positivity (TTP) read-out was converted to log_10_ CFU using stock standard curves of TTP against inoculum volume and CFU. Results are presented as growth ratio which is calculated as log_10_ CFU of sample/log_10_ CFU of control.

#### 2.3.5 Assay harmonisation

Steps were taken to ensure consistency across sites as far as possible for the harmonisation experiments, including use of the same batch of BCG Pasteur stock preparation diluted in the same series of dilutions at each site, circulating aliquots of the same cryopreserved PBMC isolated at one site, and exchange of protocols with specific details for each experiment. Operator training was conducted between sites at the start of the project in the standard in-tube protocol.

### 2.4 Cell viability studies

Cell viability at the end of the 96 h culture period was quantified by trypan blue exclusion. A 0.4% solution of trypan blue (Sigma Aldrich, MO, USA) was prepared in buffered isotonic salt solution (pH 7.2–7.3) and 0.1 ml of trypan blue stock solution added to 0.1 ml PBMC. A haemocytometer slide was loaded and examined under a microscope. The percentage of viable cells was calculated as [1-(number of blue staining cells ÷ number of total cells)] x 100, and corrected for the dilution factor.

### 2.5 IFN-γ ELISA

IFN-γ concentration in MGIA culture supernatants at the end of the 96 h period was determined using the Quantikine® ELISA kit (R&D Systems, Inc. MN, USA) according to the manufacturer's instructions. Briefly, samples were diluted 1:1 with assay diluent and incubated for 2 h at room temperature before aspirating each well and washing 4 times using wash buffer. 200 μl of human IFN-γ conjugate was added to each well and incubated for 2 h at room temperature followed by 4 washes with wash buffer. Substrate solution (200 μl) was added to each well and incubated for 30 min in the dark at room temperature, followed by 50 μl of stop solution. The optical density (OD) of each well was determined using a microplate reader set to 450 nm. Duplicate readings were averaged and the average zero optical density subtracted. A standard curve was generated to convert OD values to concentration in pg/ml.

### 2.6 Statistics

Data was analysed using GraphPad Prism v.7 and IBM SPSS v.25. Normality of data was determined using a Shapiro-Wilk test. For parametric data with multiple groups, a one-way ANOVA or repeated-measured ANOVA was conducted followed by a Dunn's post-test. For comparisons between two groups of normally-distributed data, a t-test or paired t-test was used. For non-parametric data with multiple groups, a Kruskal-Wallis or Friedman test was conducted followed by a Dunn's post-test. For comparisons between two groups of non-parametric data or small sample size, a Mann Whitney or Wilcoxon matched-pairs signed rank test was conducted. A Spearman's rank correlation was used to determine associations between two different measures. For determination of intra-assay precision, intermediate (inter-assay) precision and inter-site reproducibility, the coefficients of variation (standard deviation/mean x 100) and intra-class correlation coefficients (two-way mixed model, consistency agreement, single measures) were calculated using raw TTP values.

## 3 Results

### 3.1 Repeatability is improved with increased cell number and increased numbers of mycobacteria

Human PBMC from 4 donors at a concentration of either 1, 3 or 5 × 10^6^ cells per culture were inoculated with either 50 or 500 CFU of BCG Pasteur in the in-tube direct PBMC MGIA conducted at three different laboratory sites. On average, intra-assay repeatability between replicate cultures was improved with increased cell input and with increased mycobacterial inoculum. The mean CVs for the replicate cultures under the different assay conditions are shown in Table 1.

**Table 1:** Repeatability is improved with increased cell number and increased mycobacterial input. Human PBMC from 4 donors at a concentration of either 1, 3 or 5 × 10^6^ cells per culture were inoculated with either 50 or 500 CFU of BCG Pasteur in the in-tube direct PBMC MGIA conducted at three different laboratory sites. Median coefficient of variation (CV) values are shown at each site and averaged across sites to give the mean CV value for each MOI condition.

CFU	Cell No. (×10^6^)	CV (%)			
		Site 1	Site 2	Site 3	Mean
500	1	2.09	4.59	5.69	4.12
	3	2.09	1.04	8.93	4.02
	5	5.00	0.55	2.23	2.59
50	1	4.65	5.89	11.75	7.43
	3	3.42	4.62	11.79	6.61
	5	1.51	3.52	13.44	6.16

### 3.2 Lysis method does not affect numbers of BCG recovered

**Fig. 1:**
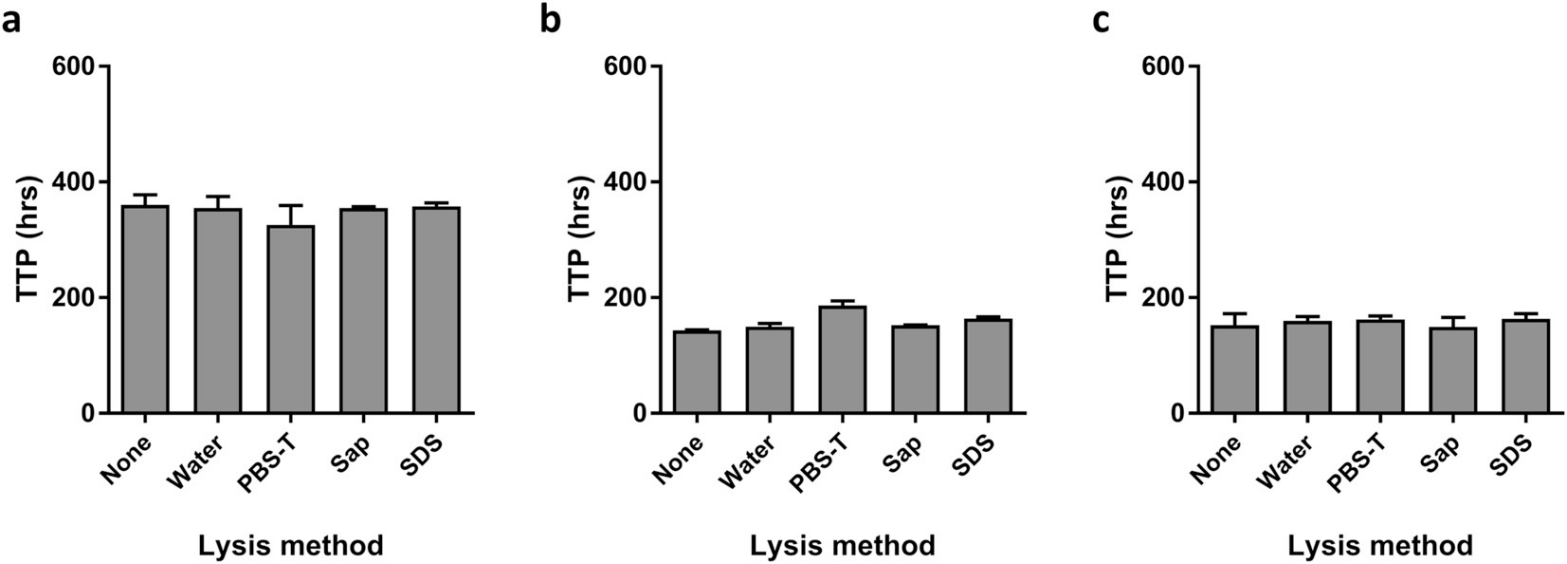
Lysis method does not affect amount of BCG recovered. Mycobacterial recovery following the direct PBMC MGIA in 2 ml screw-cap tubes was assessed using five different cell lysis conditions: none, sterile water, Phosphate Buffered Saline (PBS) with Tween 20 (PBS-T), 0.2% Saponin (Sap), and 0.067% Sodium Dodecyl (lauryl) Sulfate (SDS) at three different laboratory sites using different donor PBMC (*n* = 2 or *n* = 3 donors per condition) at each site (site 1 = a, site 2 = b, site 3 = c). Data are expressed as median values with interquartile range. TTP = time to positivity by BACTEC MGIT.

Quantification of BCG at the end of the 96 h co-culture period is a critical step in assessing mycobacterial growth inhibition. Intracellular BCG is released by lysis of host cells before addition of the cultures to BACTEC MGIT tubes using sterile water (Fletcher et al., 2013). We compared mycobacterial recovery under 5 different cell lysis conditions: (1) None, (2) Sterile water, (3) Phosphate Buffered Saline (PBS) with Tween 20, (4) 0.2% Saponin and (5) 0.067% Sodium Dodecyl (lauryl) Sulfate (SDS) across three different laboratory sites. While an in-house BCG Pasteur stock was used at site 1, sites 2 and 3 used two different batches of the same stock provided by Aeras. BCG recovery was comparable across conditions, including no lysis, at all sites (Fig. 1a–c).

### 3.3 Application of preliminary protocol to relevant clinical samples

**Fig. 2:**
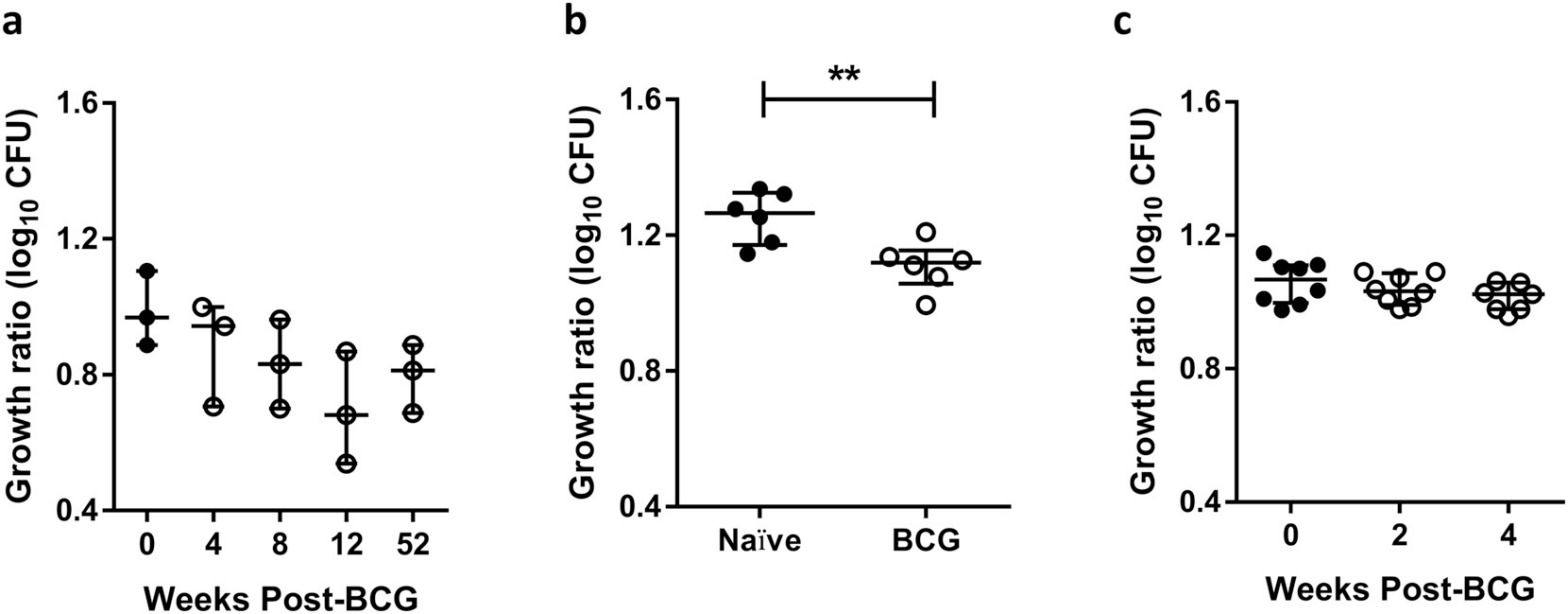
Application of preliminary SOP to relevant clinical samples. The preliminary MGIA protocol (1 × 10^6^ PBMC, 500 CFU BCG in 2 ml screw-cap tubes with no lysis) was applied to relevant clinical samples at each of the three sites. At site 1, samples were available from 3 volunteers at baseline and weeks 4, 8, 12 and 52 post-BCG vaccination (a). At site 2, samples were available from 6 BCG-naïve and 6 historically BCG-vaccinated volunteers (b). At site 3, samples were available from 8 volunteers at baseline and weeks 2 and 4 post-BCG vaccination (c). Growth ratio = log_10_(CFU of sample/CFU of control). Each point represents the mean of 2 technical co-culture replicates of the same sample where filled circles are BCG unvaccinated and open circles are BCG vaccinated, and lines represent the group median values with interquartile range. For (a and c), a Friedman test was performed followed by Dunn's post-test for multiple comparisons; there were no statistically significant differences between time-points. For (b), a Mann-Whitney *U* test was performed, where ** indicates a *p*-value of <.01.

The preliminary MGIA protocol (1 × 10^6^ PBMC and 500 CFU of BCG in 2 ml screw-cap tubes with no lysis at the end of the culture period) was applied to relevant clinical samples at each of the three sites. At site 1, samples were available from 3 volunteers at baseline and weeks 4, 8, 12 and 52 post-BCG vaccination. There was a trend towards improved control of mycobacterial growth following BCG vaccination up to week 12, but this was not statistically significant (Fig. 2a). At site 2, samples were available from 6 BCG-naïve and 6 historically BCG vaccinated volunteers. Historically vaccinated volunteers demonstrated significantly improved control of mycobacterial growth compared with naïve volunteers (*p* = .001, Mann-Whitney *U* test, Fig. 2b). At site 3, samples were available from 8 volunteers at baseline and weeks 2 and 4 post-BCG vaccination. There was a slight trend towards improved control of mycobacterial growth following BCG vaccination, but this was not statistically significant (Fig. 2c). These findings led to efforts to further optimise the protocol by investigating the use of static 48-well plates in place of rotating screw-cap tubes.

### 3.4 Cell viability and IFN-γ production is improved by co-culturing in static 48-well plates compared with rotating screw-cap tubes

**Fig. 3:**
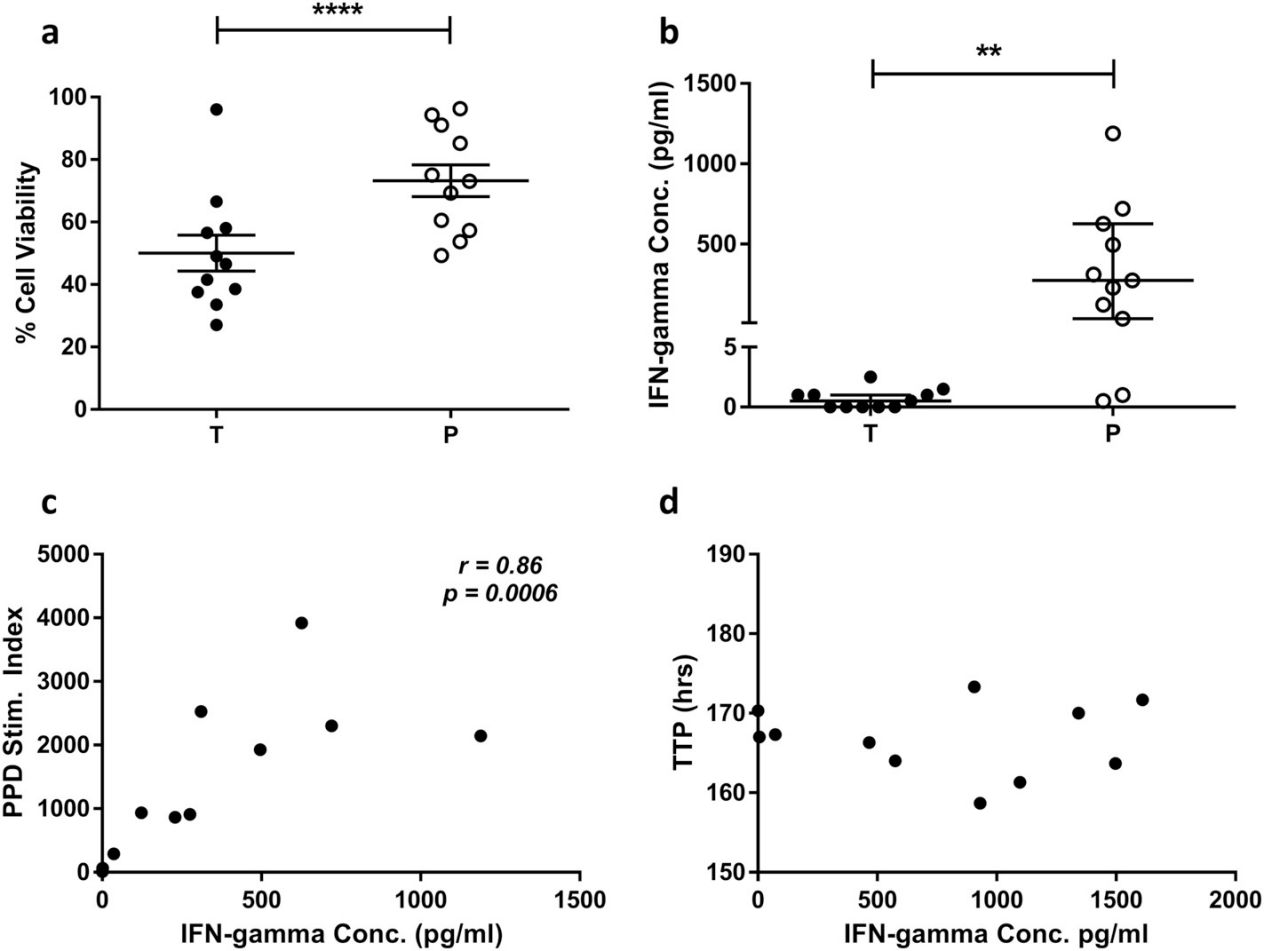
Cell viability and IFN-γ production is improved by co-culturing in static 48-well plates compared with rotating screw-cap tubes. 3 × 10^6^ human PBMC from 12 donors (one of which was excluded due to poor cell recovery post-thawing) were co-cultured with BCG Pasteur in either sealed rotating screw-cap tubes (T, filled circles) or static 48-well plates with CO_2_ (P, open circles). At the end of the 96 h culture period, cell viability was assessed by trypan blue exclusion (a), and IFN-γ concentration in the culture supernatants was measured by ELISA (b). The association between IFN-γ concentration in the MGIA culture supernatants and volunteer PPD status, as measured by PPD stimulation index (ratio of IFN-γ concentration produced by cells cultured for 72 h in the presence of PPD and those cultured without antigen), was determined for the same 12 donors (c). Mycobacterial growth in the MGIA was also measured using the 48-well plate protocol and the association with IFN-γ concentration in the 96 h supernatants was determined (d). TTP = time to positivity by BACTEC MGIT. Each point represents the mean of 2 or 3 technical co-culture replicates of the same sample and lines represent the group mean values with SEM. For (a) and (b), a Wilcoxon matched-pairs signed rank test was performed, where ** indicates a *p*-value of <.01, and **** indicates a *p*-value of <.0001. For (c) and (d), a Spearman's correlation was performed.

3 × 10^6^ human PBMC from 12 donors (one of which was excluded due to poor cell recovery post-thawing) were co-cultured with ~500 CFU of BCG Pasteur in either sealed rotating screw-cap tubes or in static 48-well plates with CO_2_. At the end of the 96 h culture period, cell viability was assessed by trypan blue exclusion, and IFN-γ production was measured by ELISA. Cell viability was significantly greater in 48-well plates compared with rotating screw-cap tubes (*p* < .0001, paired *t*-test, Fig. 3a). There was a significant correlation between cell viability in screw-cap tubes and 48-well plates (*r* = 0.85, *p* = .0007, Spearman's correlation), but no correlation between cell viability and mycobacterial growth in the MGIA (*r* = −0.04, *p* = .92, Spearman's correlation). IFN-γ concentrations were significantly higher in the supernatants of co-cultures in 48-well plates compared with those in screw-cap tubes (*p* = .003, Wilcoxon matched-pairs signed rank test, Fig. 3b), and there was a significant correlation between IFN-γ concentration and cell viability (*r* = 0.47, *p* = .03, Spearman's correlation). There was also a significant correlation between IFN-γ concentration in the MGIA supernatant and volunteer PPD status as measured by PPD stimulation index (*r* = 0.86, *p* = .0006, Spearman's correlation, Fig. 3c), but no association between IFN-γ concentration and mycobacterial growth in the MGIA (*r* = −0.12, *p* = .73, Spearman's correlation, Fig. 3d).

### 3.5 Repeatability, intermediate precision and inter-site reproducibility is improved by co-culturing in static 48-well plates compared with rotating screw-cap tubes

Human PBMC from 12 donors were assessed by MGIA with co-cultures in either sealed rotating screw-cap tubes or in static 48-well plates with CO_2_. Each sample was assayed in triplicate to assess intra-assay repeatability and the experiment repeated twice on different days to assess intermediate (inter-assay) precision. This process was repeated twice across three different sites to evaluate inter-site reproducibility.

**Fig. 4:**
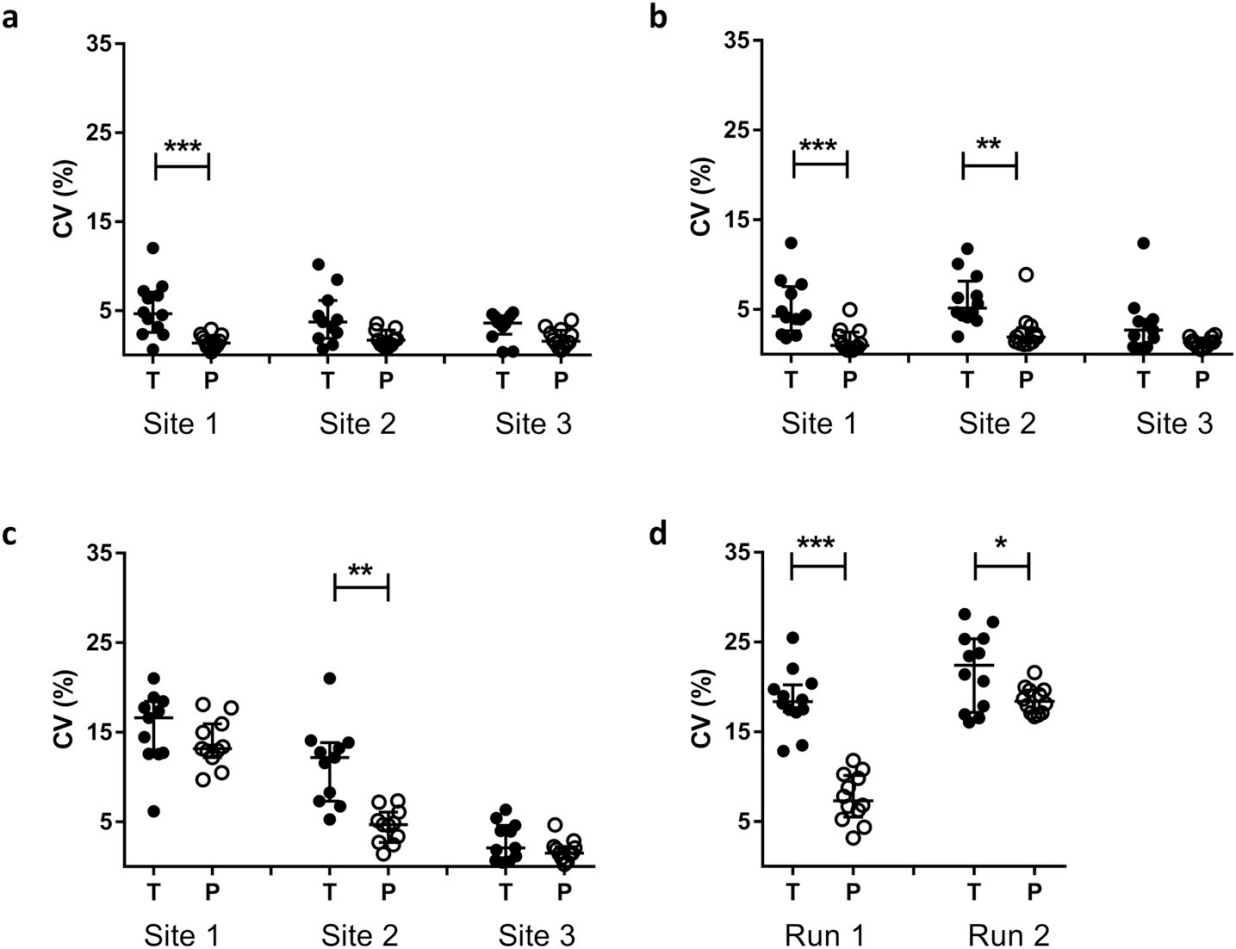
Repeatability, intermediate precision and inter-site reproducibility is improved by co-culturing in static 48-well plates compared with rotating screw-cap tubes. Human PBMC from 12 donors were co-cultured with BCG Pasteur in either sealed rotating screw-cap tubes using 1 × 10^6^ PBMC per culture (T, filled circles) or static 48-well plates with CO_2_ using 3 × 10^6^ PBMC per culture (P, open circles). At the end of the 96 h culture period, the TTP was determined using the BACTEC MGIT system. Each sample was assayed in triplicate to assess intra-assay repeatability in two different assay runs (a) and (b) and the experiment repeated twice on different days to assess intermediate (inter-assay) precision (c). The same sample set was tested at three different laboratory sites in two separate runs to evaluate inter-site reproducibility (d). Each point represents the mean of 2 or 3 technical co-culture replicates of the same sample and lines represent the group median values with interquartile range. A Wilcoxon matched-pairs signed rank test was performed between groups, where * indicates a *p*-value of <.05, ** indicates a *p*-value of <.01, and *** indicates a *p*-value of <.001. CV = coefficient of variation.

In run 1, intra-assay repeatability between replicate cultures, as measured by CV, was significantly improved when using 48-well plates compared with rotating screw-cap tubes at site 1 (*p* = .001, Wilcoxon matched-pairs signed rank test, Fig. 4a). At site 2, a significant outlier was identified using the maximised normalised residual (Grubb's) test. Following exclusion of this outlier, repeatability was significantly greater using 48-well plates compared with rotating screw-cap tubes at this site (*p* = .04, Wilcoxon matched-pairs signed rank test). In run 2, intra-assay repeatability was significantly improved when using 48-well plates compared with rotating screw-cap tubes at sites 1 and 2 (*p* = .001 and *p* = .003 respectively, Wilcoxon matched-pairs signed rank test, Fig. 4b). There was a trend towards improved repeatability at site 3 across both runs but this was not statistically significant. The mean intra-class correlation coefficients (ICCs) and CVs for the replicate cultures under the different assay conditions are shown in Table 2.

**Table 2:** Repeatability is improved by co-culturing in static 48-well plates compared with rotating screw-cap tubes. Human PBMC from 12 donors were co-cultured with BCG Pasteur in either sealed rotating screw-cap tubes or in static 48-well plates with CO_2_. At the end of the 96 h culture period, the amount of BCG remaining was quantified using the BACTEC MGIT system. Each sample was assayed in triplicate to assess intra-assay repeatability and the experiment repeated twice on different days across three different sites. Intraclass correlation coefficient (ICC) and median coefficient of variation (CV) values are shown at each site and averaged across sites for each condition.

	ICC				CV (%)			
	Site 1	Site 2	Site 3	Mean	Site 1	Site 2	Site 3	Mean
Screw-cap tubes	0.35	0.58	0.33	0.42	4.44	3.22	4.38	4.01
48-well plates	0.81	0.40	0.66	0.62	1.17	1.51	1.74	1.47

Intermediate (inter-assay) precision between experiments was determined by conducting the direct PBMC MGIA using the same sample set at the same site on two different days (henceforth referred to as ‘runs’). Intermediate precision, as measured by CV, was significantly improved when using 48-well plates compared with rotating screw-cap tubes at site 2 (*p* = .002, Wilcoxon matched-pairs signed rank test, Fig. 4c). There was a trend towards improved intermediate precision with 48-well plates at sites 1 and 3 but this was not statistically significant. The mean ICCs and CVs for the two runs under the different assay conditions are shown in Table 3. Inter-site reproducibility was determined by conducting the direct PBMC MGIA using the same sample set at three different laboratories repeated in two separate runs. Inter-site reproducibility, as measured by CV, was significantly improved when using 48-well plates compared with rotating screw-cap tubes in both runs 1 and 2 of the experiment (*p* = .0005 and *p* = .02 respectively, Wilcoxon matched-pairs signed rank test, Fig. 4d). The mean ICCs and CVs across the three sites under the different assay conditions are shown in Table 4.

**Table 3:** Inter-assay precision is improved by co-culturing in static 48-well plates compared with rotating screw-cap tubes. Human PBMC from 12 donors were co-cultured with BCG Pasteur in either sealed rotating screw-cap tubes or in static 48-well plates with CO_2_. At the end of the 96 h culture period, the amount of BCG remaining was quantified using the BACTEC MGIT system. The experiment was repeated twice on different days across three different sites. Intraclass correlation coefficient (ICC) and median coefficient of variation (CV) values are shown at each site and averaged across sites for each condition.

	ICC				CV (%)			
	Site 1	Site 2	Site 3	Mean	Site 1	Site 2	Site 3	Mean
Screw-cap tubes	0.46	0.48	0.11	0.35	16.62	12.18	2.09	10.30
48-well plates	0.47	0.71	0.44	0.54	13.17	4.68	1.49	6.45

**Table 4:** Inter-site reproducibility is improved by co-culturing in static 48-well plates compared with rotating screw-cap tubes. Human PBMC from 12 donors were co-cultured with BCG Pasteur in either sealed rotating screw-cap tubes or in static 48-well plates with CO_2_. At the end of the 96 h culture period, the amount of BCG remaining was quantified using the BACTEC MGIT system. The experiment was repeated twice on different days across three different sites. Intraclass correlation coefficient (ICC) and median coefficient of variation (CV) values between sites averaged across runs are shown.

	ICC	CV (%)
Screw-cap tubes	0.20	20.39
48-well plates	0.33	12.84

**Supplementary Fig. 1:**
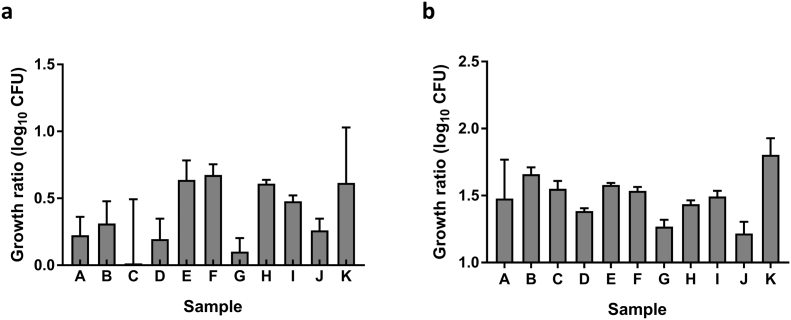
MGIA mycobacterial control using rotating screw-cap tubes *vs.* static 48-well plates. Cryopreserved PBMC were taken from healthy Dutch adult volunteers and the direct PBMC MGIA performed using either 2 ml screw-cap tubes with 1 × 10^6^ PBMC per culture (a), or 48-well plates with 3 × 10^6^ PBMC per culture (b). Data are expressed as median values with interquartile range. Growth ratio = log_10_(CFU of sample/CFU of control).

There was no association between mycobacterial growth measured using the in-tube protocol and the 48-well plate protocol across any of the three sites, as indicated by negative ICC values (data not shown). However, this sample set was homogeneous with similar levels of growth control across volunteers (range = 0.29 and 0.13 log_10_ CFU for the in-tube and 48-well plate protocols respectively), resulting in a low signal-to-noise ratio. When tested in an additional sample set with a broader dynamic range (range = 0.66 and 0.59 log_10_ CFU respectively), a similar pattern of control across the individuals was observed in a direct comparison of the two protocols described here with an ICC of 0.54 (moderate agreement) (Supplementary Fig. 1a–b).

### 3.6 Assay harmonisation using relevant clinical samples

**Fig. 5:**
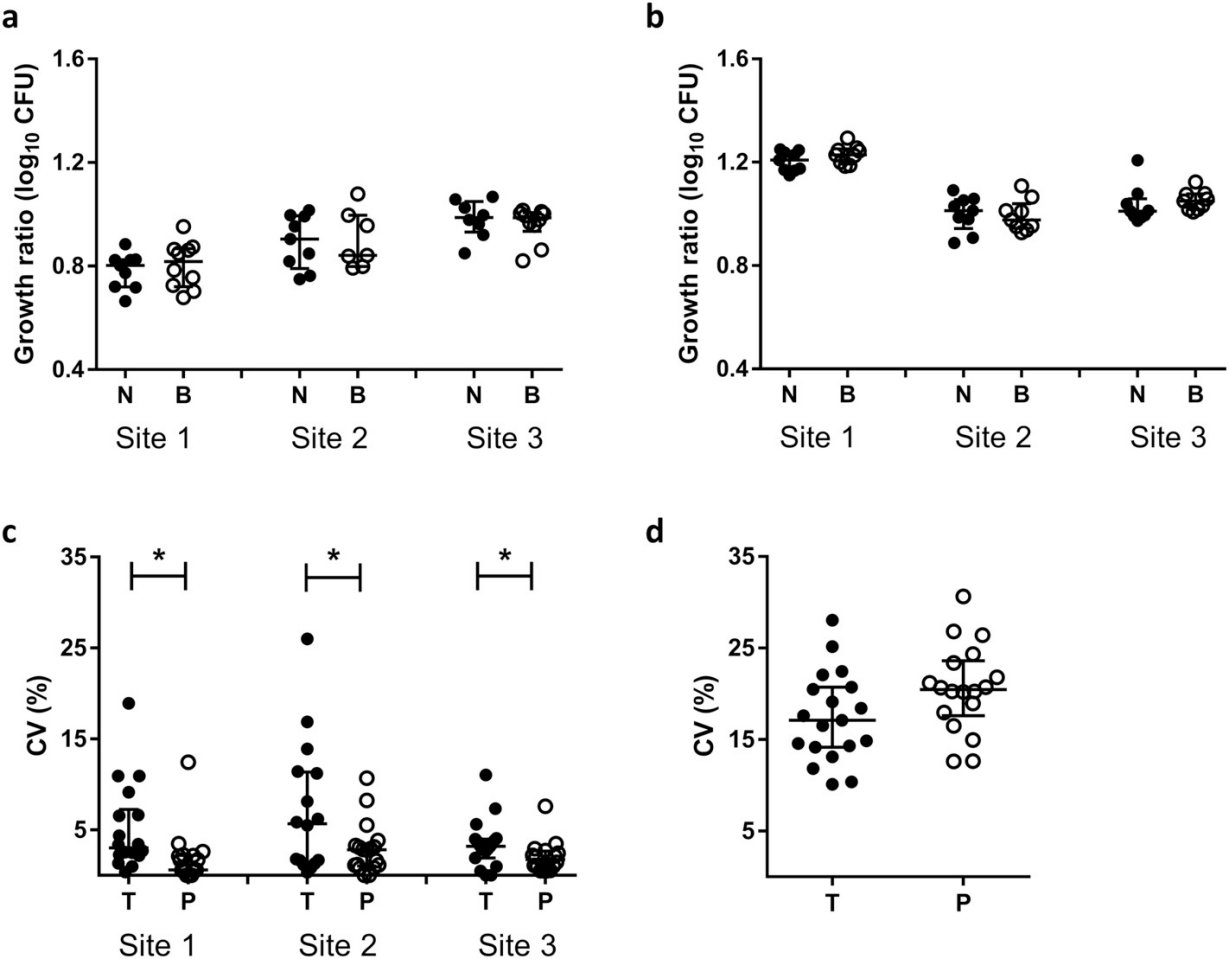
Assay harmonisation using relevant clinical samples I. The MGIA using either 2 ml screw-cap tubes with 1 × 10^6^ PBMC per culture (a) or 48-well plates with 3 × 10^6^ PBMC per culture (b) was applied to a shared set of human PBMC from 20 donors; 10 of which were BCG-naïve (N, filled circles) and 10 of which were historically BCG vaccinated (B, open circles). Each sample was assayed in duplicate to assess intra-assay repeatability (c) and the experiment conducted once at each of three different laboratories to measure inter-site reproducibility (d). Growth ratio = log_10_(CFU of sample/CFU of control). Each point represents the mean of 2 or 3 technical co-culture replicates of the same sample and lines represent the group median values with interquartile range. A Wilcoxon matched-pairs signed rank test was performed between groups, where * indicates a *p*-value of <.05. CV = coefficient of variation.

Cryopreserved cells were taken from adult volunteers who were either BCG naïve or historically BCG vaccinated and the direct PBMC MGIA performed in duplicate across three different sites. There was no overall difference in MGIA outcome between naïve and historically vaccinated volunteers observed at any of the three sites using either the in-tube (Fig. 5a) or 48-well plate (Fig. 5b) protocols. The intra-assay repeatability, as measured by CV, was significantly improved when using 48-well plates compared with rotating screw-cap tubes at sites 1, 2 and 3 (*p* = .02, *p* = .03 and *p* = .01 respectively, Wilcoxon matched-pairs signed rank test, Fig. 5c). Inter-site reproducibility was not different between assay conditions (Fig. 5d).

**Fig. 6:**
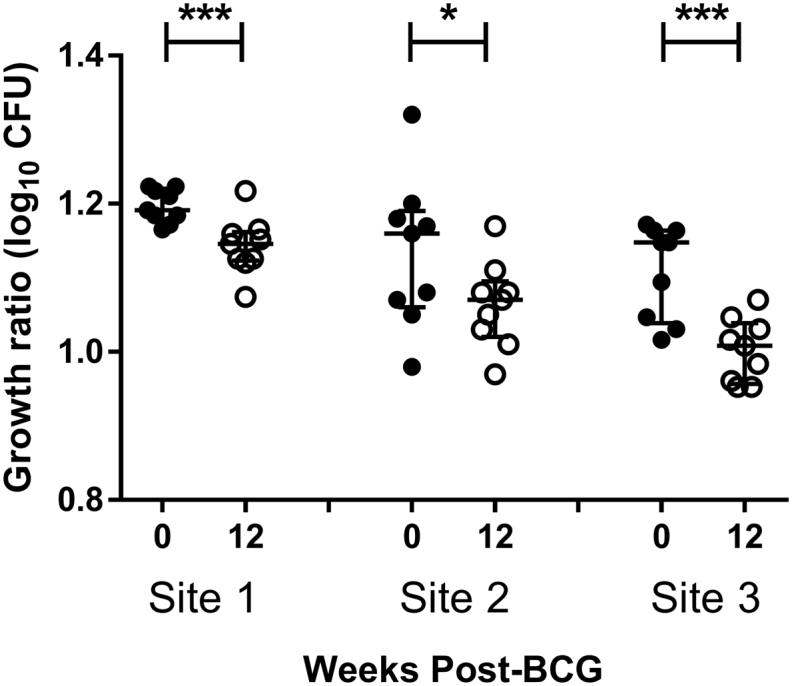
Assay harmonisation using relevant clinical samples II. Cryopreserved cells were taken from 9 UK adult volunteers at baseline and 12 weeks post-BCG vaccination and the direct PBMC MGIA performed across three different sites using the optimised 48-well plate protocol only. Growth ratio = log_10_(CFU of sample/CFU of control). Each point represents a sample cultured in singlicate and lines represent the mean values with SEM. Data was found to be normally distributed using a Shapiro-Wilk test so a paired t-test was performed, where * indicates a *p*-value of <.05 and *** indicates a *p*-value of <.001.

In a second round of experiments, cryopreserved cells were taken from UK adult volunteers at baseline and 12 weeks post-BCG vaccination and the direct PBMC MGIA performed across three different sites using the optimised 48-well plate protocol only. In order to validate detection of a ‘positive response’, volunteer samples were selected based on a) demonstrating improved control of mycobacterial growth following BCG vaccination as measured at the trial site and b) having sufficient availability of cryopreserved PBMC to conduct a comparison across three different sites. 9 volunteers fulfilled these criteria and this sample set was circulated for MGIA analysis. A significant improvement in mycobacterial growth inhibition following BCG vaccination was observed at all three sites (*p* = .0007, *p* = .01 and *p* = .0002 for sites 1, 2 and 3 respectively, paired *t*-test, Fig. 6a–c). Sample replicates were not assayed in this single-run experiment due to cell availability, and therefore it was not possible to calculate intra-assay repeatability or intermediate precision. However, inter-site reproducibility was <20% CV for both baseline and day 84 samples (data not shown).

### 3.7 EURIPRED direct PBMC MGIA SOP

The detailed optimised Standard Operating Procedure (SOP) for the direct PBMC MGIA, produced as a result of the EURIPRED project, is available online at:

http://www.euripred.eu/information-trainings/sops-assay-harmonisation.html

## 4 Discussion

While several MGIAs have been described in the literature for use in mice, cattle and humans (reviewed in Tanner et al., 2016), none has been systematically assessed for reproducibility or standardised across laboratories. The aims of the study described were to optimise, standardise and harmonise the direct PBMC MGIA to ensure that comparable results are generated by TB vaccine studies conducted at different organisations and settings.

The finding that intra-assay repeatability is improved by increasing cell concentration or mycobacterial input is consistent with previous work by Zelmer et al. (2016) using the direct mouse splenocyte MGIA (Zelmer et al., 2016). This study determined that the effect size between naïve and BCG vaccinated mice was also greatest using the highest cell and mycobacterial inputs (Zelmer et al., 2016). Ideally the magnitude of growth inhibition in clinical samples following vaccination would be compared under different multiplicity of infection (MOI) conditions as in the mouse study but this was not possible due to limited cell availability. Based on a rationalised compromise between our reproducibility findings and the limitations of clinical sample collection, we selected 3 × 10^6^ PBMC and 500 CFU of BCG Pasteur as the human assay conditions for further evaluation. Applying different lysis agents (or indeed removing the lysis step) did not impact BCG recovery, likely due to the fact that end-of-culture samples are added to BACTEC MGIT tubes containing Middlebrook 7H9 broth in which cells would naturally lyse, releasing intracellular mycobacteria for quantification. This outcome was consistent across the three sites despite the use of different BCG Pasteur stocks between sites 1 and 2/3, and different batches of the same stock between sites 2 and 3 in this experiment, which accounts for the observed inter-site variation in TTP. As such we selected the ‘no lysis’ condition for the in-tube protocol to avoid introducing unnecessary processing steps and enhance technical simplicity.

When a preliminary in-tube protocol incorporating the ‘no lysis’ condition was applied to clinical samples from the three laboratory sites, there was a significant effect of BCG vaccination detected at one site, and trends at the other two. However, ability to observe statistically significant responses was likely limited by small sample size at site 1 (*n* = 3) and early post-vaccination time-points at site 3 (weeks 2 and 4). It has previously been shown that the peak of response following primary BCG vaccination in this assay occurs at 4–12 weeks post-vaccination, but may vary from individual to individual making it difficult to reach statistical significance at a given time-point (Fletcher et al., 2013; Joosten et al., 2018). The delay may be due to the time taken to mount a vaccine-specific adaptive immune response, although it remains to be determined whether this is the underlying mechanism responsible for the observed growth inhibition (Jensen et al., 2017; Joosten et al., 2018). The lack of sensitivity to detect a vaccine response may also be because the protocol used was not at this stage fully optimised for cell viability and functionality, as improved control of mycobacterial growth following BCG vaccination has been described in several other human MGIA studies (Cheng et al., 1988; Hoft et al., 2002; Fletcher et al., 2013; Smith et al., 2016).

Due to this observed low sensitivity to detect a BCG vaccine response and concerns regarding cell viability in the murine direct MGIA using screw-cap tubes (Jensen et al., 2017), we compared the standard in-tube protocol to a static 48-well plate adaptation described in mouse splenocytes by Yang et al. (2016) (Yang et al., 2016). Co-culturing in 48-well tissue culture plates significantly improved total cell viability by a mean of 23% at the end of the 96 h period. This is likely due to availability of CO_2_ and lack of mechanical perturbation, which although necessary to prevent the separation of whole blood cultures, is damaging to the more fragile previously cryopreserved PBMC. Indeed, a similar phenomenon was observed by Jensen et al. (2017) using the direct mouse splenocyte MGIA whereby enrichment of the culture medium by addition of nutrients and incubation in screw-cap tubes without rotation increased cell viability from a mean of 21% to 46% (Jensen et al., 2017).

Interestingly, viability was considerably better for human PBMC (mean of 50% using the screw-cap tube protocol and 73% using the 48-well plate protocol) compared with the values for murine splenocytes. Murine splenocytes are widely considered more delicate than PBMC, necessitating the use of fresh samples and suggesting that the human MGIA may be more robust. However, other differences between these studies may also have contributed including the use of virulent *M.tb* Erdman in the murine study compared with BCG Pasteur, and different MOIs (5 × 10^6^ cells with 50 CFU *M.tb* compared with 3 × 10^6^ cells with 500 CFU BCG). Total cell viability did not correlate with control of mycobacterial growth suggesting that cells dying over the 96 h incubation period may not be those with effector functions. The consistency in outcomes observed in a direct comparison of both assay methods (Supplementary Fig. 1) supports this suggestion, although such relationships are likely to be complex in a functional sum-of-the-parts assay and it is unclear at what point during the culture period any given cell type will mediate its effect.

IFN-γ production in response to mycobacterial infection, a measure of cell functionality, was almost absent in supernatants at the end of the co-culture period using screw-cap tubes. However, there was a significant increase in IFN-γ concentrations measured following the 48-well plate protocol. This is likely related to improved cell viability, as supported by the significant association observed between these two measures. The concentration of IFN-γ did not correlate with mycobacterial growth control, which is consistent with previous reports using this and other MGIAs (Hoft et al., 2002; Kampmann et al., 2004; Fletcher et al., 2013; Joosten et al., 2018). A lack of association suggests that the assay is measuring parameters different from IFN-γ production alone, supporting its use as an additional tool for assessing vaccine immunogenicity. Use of 48-well plates also improved the repeatability, intermediate precision and reproducibility compared with screw-cap tubes - a finding that was replicated in the assay harmonisation phase of the project using cells from BCG naïve and historically vaccinated volunteers. This may be a direct result of improved cell viability and functionality, but it should also be noted that a lower cell number was used in the screw-cap tubes for these experiments (1 × 10^6^ PBMC compared with 3 × 10^6^ PBMC used in the 48-well plate protocol) to align with the standard in-tube protocol described in previous studies (Fletcher et al., 2013; Smith et al., 2016; Tanner et al., 2017; Joosten et al., 2018).

Tuomela et al. (2005) suggest that for assay validation a CV of <50% is acceptable variation for the measurement of a bacterial target in a cell-based assay (Tuomela et al., 2005). Overall the precision and reproducibility of the direct PBMC MGIA was remarkably high for a cell-based functional assay. We found that repeatability (intra-assay precision) was <15% and < 5% CV for the screw-cap tube and 48-well protocols respectively. Intermediate precision (inter-assay precision) was <25% CV, while inter-site reproducibility was <30% CV. Variability between sites is expected to be greater than between assay runs at the same site which in turn is expected to be greater than between replicates within a single run, as each level involves an ever-greater number of opportunities to introduce variability. In a recent multi-site study of technology transfer, CVs for inter-site reproducibility for established standardised immunological assays such as ELISpot and intracellular cytokine staining (ICS) were approximately 50% even after side-by-side training (Smith et al., 2017), suggesting the direct MGIA for both protocols to be a more consistent assay for immunological monitoring of vaccination studies than the widely-used cytokine assays. It should be noted, however, that cells were isolated and cryopreserved at a single central facility, and thus potential effects of differential PBMC processing and storage are not taken into account.

One criticism of using CV for such analysis is that the signal-to-noise ratio is not taken into account. CV values may appear artificially low because the range of values is small (including across the sample set), or artificially high due to systematic differences. ICC compares within-sample variability (between replicates within an assay run or between runs) with between-sample variability and therefore arguably offers a more stringent and meaningful measure. ICC can also be applied using a ‘consistency’ rather than ‘absolute’ agreement, which takes into account systematic differences between operators. Thus ICC was also calculated where sample size permitted. Using the criteria described by Landis and Koch (1977), the ICC for repeatability (intra-assay precision) was ‘moderate’ for the in-tube protocol and ‘substantial’ for the 48-well plate protocol. The ICC for intermediate (inter-assay) precision was ‘fair’ and ‘moderate’; while the ICC for inter-site reproducibility was ‘slight’ and ‘fair’ for the in-tube and 48-well plate protocols respectively. Inter-site reproducibility may be improved by side-by-side training of operators in the newly-defined SOP (Smith et al., 2017). Standardising the BCG stock between sites also had a considerable impact on inter-site reproducibility; in the first experiment where different BCG Pasteur stocks were used between sites 1 and 2/3 there was a 2× difference between TTPs that was improved in subsequent experiments where a common stock was shared.

In the final assay harmonisation phase of the project, we applied our optimised assay conditions and standardised SOP (3 × 10^6^ cells, 500 CFU BCG, 48-well plates with CO_2_) to two shared sets of clinical trial samples. In the first cohort, which consisted of BCG naïve and historically vaccinated volunteers, we did not observe a difference between the two groups at any of the three sites or using either protocol. That this finding was consistent across all three sites and both protocols suggests that this is due to biological rather than technical factors. These volunteers were vaccinated at various times in the past (up to 40 years ago) and as such we may not expect to detect an effect of vaccination, particularly given that BCG efficacy has been shown to reduce over time in some studies (Mangtani et al., 2018), and a previous study found the MGIA response to have waned by 24 weeks post-vaccination (Fletcher et al., 2013). However, given that a significant effect was observed between naïve and historically-vaccinated individuals in an earlier experiment (Fig. 2b), this result may be due to the particular donors used, as BCG is known to confer variable efficacy *in vivo*.

A second clinical trial cohort was also tested where samples were available from baseline and 12 weeks post-vaccination. A significant improvement in mycobacterial control was observed across all three sites following vaccination using these samples, consistent with reports of a BCG vaccine effect in several previous human MGIA studies (Cheng et al., 1988; Kampmann et al., 2004; Fletcher et al., 2013; Smith et al., 2016; Joosten et al., 2018) and high efficacy of BCG vaccination in this (UK) population (Fine, 1995). In addition to using samples from recently BCG-vaccinated volunteers, a further important difference between this and the previous harmonisation experiment was the addition of autologous time-point matched serum rather than pooled human serum to the MGIA co-cultures. Although data are not available from the same samples using pooled human serum, it has previously been demonstrated that post-BCG-vaccination sera enhance mycobacterial growth inhibition *via* antibody-mediated mechanisms (de Vallière et al., 2005; Chen et al., 2016), and we suggest that use of autologous serum offers a more representative *ex vivo* approach. This may be of particular importance when assessing antibody-inducing vaccine candidates, and merits further investigation in the context of this assay. We therefore recommend the use of autologous serum where available. If pooled human serum is used then preparation should be standardised; preferably using the same batch of commercially purchased human AB serum which has been heat inactivated at 56 °C for 30 min prior to storing at −20 °C, and thawed at room temperature before addition to co-cultures.

In conclusion, we have taken steps to optimise the human direct PBMC MGIA and assess assay precision at multiple levels. We have defined recommended assay conditions and noted that co-culturing in 48-well plates resulted in improved cell viability, IFN-γ production and reproducibility between replicates, assay runs and sites. Based on these findings, we produced a standardised operating procedure (SOP) using 3 × 10^6^ cells in 48-well plates with C0_2_ which was applied to relevant clinical samples. These conditions resulted in improved reproducibility compared with a previously-described in-tube protocol using 1 × 10^6^ cells, increasing the signal-to-noise ratio and ability to detect a vaccine-induced response. However, we demonstrated that where dynamic range is sufficient, there is moderate agreement between outcomes from the two protocols, and that reproducibility of the in-tube protocol is still well within acceptable limits for a cell-based functional assay (Tuomela et al., 2005). As such, the in-tube protocol may be applied where cell number is limiting and biological effects strong, and can be used to further dissect the mechanism of mycobacterial growth control (Smith et al., 2016; Tanner et al., 2017; Joosten et al., 2018). The optimisation and harmonisation efforts described here resulted in demonstrable consistency in outcomes between three laboratory sites using clinically-relevant samples. This is a critical step in assay development to ensure that comparable information can be extracted from TB vaccine studies conducted across different organisations. Additional work is now required to further optimise and biologically validate the direct MGIA by demonstrating a correlation with *in vivo* protection from mycobacterial challenge, and to evaluate the performance of the assay in trials of novel TB vaccine candidates.

The following are the supplementary data related to this article.
